# The effect of hydro-alcoholic extract of *Achillea millefolium* on appetite hormone in rats

**Published:** 2017

**Authors:** Mohsen Nematy, Mohsen Mazidi, Atefeh Jafari, Sara Baghban, Hasan Rakhshandeh, Abdolreza Norouzy, Habibollah Esmaily, Leila Etemad, Michael Patterson, Amir Hooshang Mohammadpour

**Affiliations:** 1 *Biochemistry and Nutrition Research Center and Department of Nutrition, Mashhad University of Medical Sciences, Mashhad, Iran *; 2 *Department of Clinical Pharmacy, School of Pharmacy, Mashhad University of Medical Sciences, Mashhad, Iran*; 3 *Pharmacological Research Center of Medicinal Plants, School of Medicine, Mashhad University of Medical Sciences, Mashhad, Iran*; 4 *Health Sciences Research Center, Department of Biostatistics and Epidemiology, School of Health, Mashhad University of Medical Sciences, Mashhad, Iran*; 5 *Pharmaceutical Research Center, Mashhad University of Medical Sciences, Mashhad, Iran*; 6 *Department of Life Sciences, University of Roehampton, London SW15 4JD*

**Keywords:** Achilleamillefolium, Appetite, Food intake, Ghrelin, Hydro-alcoholic extract

## Abstract

**Objective:**

*Achillea millefolium *(*A. millefolium*) is known as an orexigenic herb in Iranian traditional medicine. In this study, the possible orexigenic effect of hydro-alcoholic extract of *A. millefolium* was investigated by measuring plasmaghrelin level.

**Materials and Methods:**

Thirty male Wistar rats were divided into five groups. Control group received water. Treatment groups received 50, 100 or 150 mg/kg of *A. millefolium* extract for 7 days via gavage. Before the intervention, daily amount of the food eaten by each rat was measured for 10 days. During the investigation, the amount of energy intake of each rat was also estimated 1, 2, 4, 6 and 24 hr after each intake, for 7 days. Later, the orexigenic dose of extract and distilled water was fed to two separate groups of 6 male Wistar rats. Plasma ghrelin level was measured 0.5, 1, 2 and 4 hrafter extract intake.

**Results:**

The change in energy intake after treatment by 50and 100mg/kg of the extract was significantly higher than other groups (p<0.001).Administration of Achillea 100mg/kg significantly (p<0.05) decreased ghrelin level one hrafter intervention but there was no significant (p>0.05) difference among control and treated group.

**Conclusion:**

This study indicated that *A. millefolium* had positive dose-related effects on appetite in rats. It seems that the orexigenicactivity of extract was not related to changes in plasma ghrelin levels.

## Introduction

Weight loss due to cachexia is a global health problem. Studies have shown that newly discovered ghrelin hormone plays an important role in prevention of this condition(Akamizu et al., 2010b ▶) . Ghrelin is a peptide consisting of 28 amino acids which is secreted from oxyntic cells of fundus and it was shown to be an endogenous ligand for the GH secretagogues receptor (GHS-R1a) (Kojima et al., 1999 ▶; Shimbara et al., 2004 ▶). This hormone was used as the first orexigenic peptide which is secreted by stomach and its main effect is considered to be food intake enhancement (Wren et al., 2001 ▶). In Iranian TraditionalM edicine, some herbs like *Achillea millefolium*, commonly known as yarrow, are implicated as appetite enhancers. However, there is not enough research evidence to prove their actual effect. Achillea millefolium is a dicot which belongs to the family *Asteraceae.* Today, *Achillea millefoliumis* found in most temperate zones such as Iran, Canada, Europe and northern China. It long has been used as a medicine by many cultures to treat loss of appetite, dyspeptic (digestive) complaints, and liver and gallbladder complaints. 

Yarrow can improve food intake through increased bile flow and stimulating the secretion of digestive enzymes (Rhiannon 2005 ▶). It seems that effective manipulation of ghrelin secretion by herbal medicines in order to improve appetite provides alternatives with lower cost and fewer side effects. There has been little knowledge about *A. millefolium* and its orexigenic effect. Also, no study to date has directly investigated the effect of this herb on plasma ghrelin level. The main issue addressed in this paper is: Does *A. millefolium* administration lead to elevated ghrelin level in rats?

## Materials and Methods

**Animals**

Thirty male Wistar rats (weight 200-220 g) were purchased from animal house of Mashhad University of Medical Sciences, Mashhad, Iran. They were kept at 22±1 °C with 12 hr/12 hrlight/dark cycle. All rats had access to food sources without any limitation. The protocol of this study was approved by the Animal Care and Ethics Committee of Mashhad University of Medical Sciences, Mashhad, Iran.

**Foods**

Laboratory animal food was provided by Javaneh Khorasan Company (Iran). It was constituted of 21% protein and 6.5 – 7% fat. Each 1000 g of food contained 2750 Kilocalories. 

**Preparation of Hydro alcoholic Extract**

*A. millefolium* was provided by herbarium of Mashhad University of Medical Sciences (Voucher Specimen No.12305). Pharmacologic parts of A. millefolium are seed, leaf and stem (Lakshmi et al., 2011 ▶). The whole plant was dried at room temperature in shadow, and then powdered. The particle size must not be greater than 4.572 cm. Alcoholic extract was obtained by maceration. The powder was soaked in 70% alcohol, as the solvent, in a tightly closed container for 3 days. The container was shaken one or two times a day. The mixture was then filtered to separate the solute using rotary evaporator. The extract was dried in oven at <40 °C. The dried extract was dissolved in distilled water to prepare relative concentrations before gavage every day. Lack of extract standardization was one of the limitations of this study.

**Experimental design**

The experiments were conducted in two parts. In the first part of study, thirty male Wistar rats were randomly selected and divided into five groups ofsix rats. Before intervention, 24-hr food intake of each rat, for 10 consecutive days, was measured. Animals had free access to food and water. To investigate the relationship between A. millefolium extract and plasma ghrelin levels, yarrow extract (50, 100 and 150 mg /kg)was administered to the groups for 7 days via gavage. The rats in the control group received 0.5 ml distilled water every day. The food consumption (in grams) by each rat was measured 1, 2, 4, 6 and 24 hrafter extract administration. The average food intake of each rat was converted to Kcal/day. The mean Kcal received during 10 days before intervention and mean Kcal received 1, 2, 4, 6 and 24 hrafter extract administration during 7 days of intervention was measured.

In the second part of the study, after administration of selected extract concentration, according to the first part, blood samples were collected at certain intervals to measure plasma ghrelin levels by ELISA method. Twelve rats were randomly selected and divided into 2 groups of 6 rats. Animals were fasted 12 hrbefore the start of the second part. Blood samples were collected from the orbital sinus with heparinized capillary tubes, to measure the base ghrelin level (time zero). For this purpose, rats were anaesthetized for a short time by ether inhalation. Later, the orexigenic dose of yarrow extract and distilled water was gavaged to the intervention and control group, respectively. General anesthesia was induced with intra-peritoneal injection of 1.5mg/kg urethane. The jugular vein was cannulated to withdraw the blood samples at 0.5, 1, 2 and 4 hr intervals after gavage. The diminished blood volume was replaced with normal saline. Blood samples were centrifuged at low temperature and acidic condition and plasma was separated. Plasma samples were stored in -20±5 ˚ C to measure the ghrelin levels. 

**Statistical analysis**

The data was expressed as Mean±SEM. Statistical analyses were performed with ANOVA followed by Tukey–Kramer test to compare the differences between means. pvalues less than 0.05 were considered as significant.

## Results

Based on our results, 24-hr calorie intake before intervention compared to that after intervention at 50, 100 and 150 mg/kg of extract showed a significant difference (p<0.001).It is notable that concentrations of 50 and 100mg/kg caused a significant increase in appetite of rats, while 150mg/kg statistically decreased it (Table 1).

**Table1:** Mean energy intake 24 hrbefore and during intervention in five groups of rats.

**Groups**	**Before intervention (Kcal)**	**During intervention (Kcal)**	**p. value**
**First case **	53.7±0.66	58.5±0.12	<0.001
**Second case**	54.4±0.19	61.8±0.23	<0.001
**Third case**	53.6±0.33	46.8±0.87	<0.001
**Control **	53.7±0.48	52.8±0.53	0.24

The first, second and third case groups received 50, 100, 150 mg/kg Achillea solution, respectively. Values are expressed as Mean±SEM (Two-way ANOVA).

*** p <0.001 compared to control group.

**Figure1:**
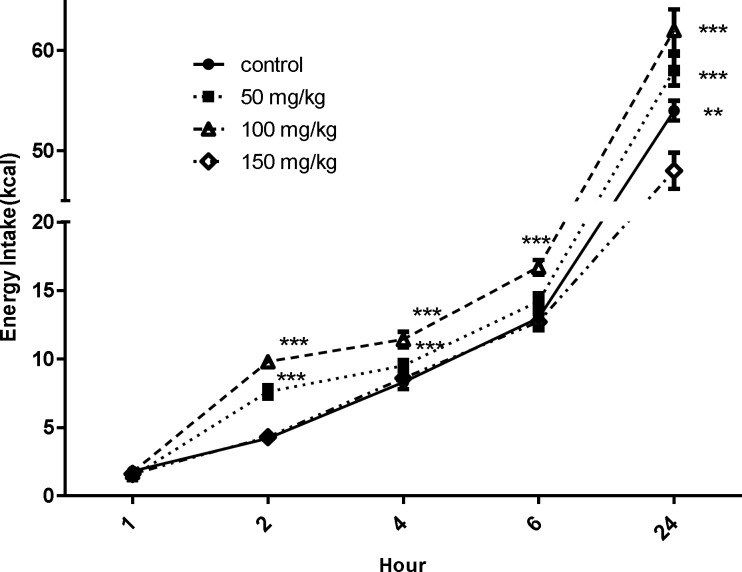
Energy intake during one, two, four, six and 24 hr after administration of yarrow extract (at different doses) or distilled water, during 7 days. Values are expressed as Mean±SEM (Two-way ANOVA). ***p < 0.001, **p<0.01 compared to control group

As it is seen in Figure 1, no statistically significant differences were found in energy intake one hour after extract administration among control and other groups (p>0.05).

The data revealed that the amount of total energy intake,two hours after extract administration in the rats receiving 50 and 100mg/kg of extract showed a significant (p<0.001) increase compared to the control group, but 150 mg/kg of extract did not result in significant difference (p>0.05) compared with control group. The amount of total energy intake, four hours after administration of extract (50 and 100mg/kg) exhibited a significant (p<0.05) increase compared to the control group. Nevertheless, 150mg/kg of extract showed no significant difference (p>0.05) compared with control group (Figure 1).

From the data in Figure 1, it is apparent that the volume of total energy intake, six hours after extract administration at a dose of 100mg/kg was significantly (p<0.001) increased compared to the control group. However, the extract at the dose of 50 mg/kg, slightly but not significantly, resulted in higher food intake by rats.

From this data, we can see that the amount of total energy intake during 24 hr after extract administration at doses of 50 and 100mg/kg displayed a significant (p<0.05) increase compared to the control group.

Comparison of amount of energy intake after extract administration at concentrations of 50 and 100mg/kg and 24 hrbefore that, showed no significant (p>0.05) difference. Because of higher energy intake in rats after extract administration at all intervals, except energy intake after one hour, at dose of 100mg/kg, this dose was chosen for the second part of the survey.

**Figure 2:**
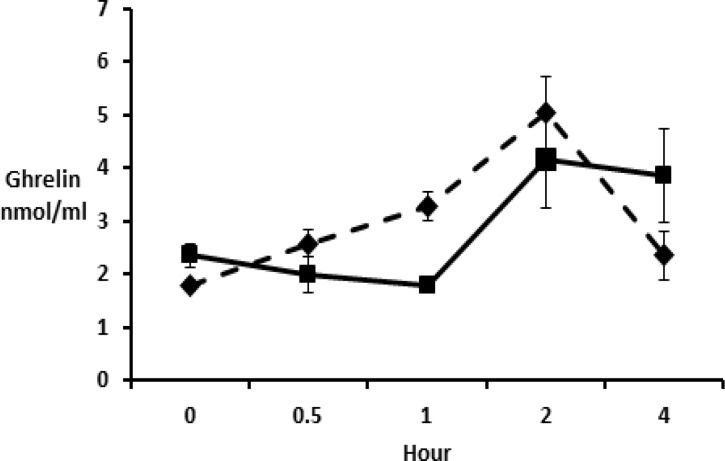
Ghrelin level 0, 0.5, 1, 2 and 4 hours after gavage. Six rats per each group received 0 (interrupted lines) and 100mg/kg (continuous line) of yarrow extract. Values are presented as Mean±SEM (Two-way ANOVA). There is no significant difference between means of ghrelin level between two groups

Here, ghrelin levelsat five time points were compared. Interestingly, administration of *Achillea* at 100mg/kg significantly (p<0.05) decreased ghrelin level one hour after administration. It is interesting to note that there was no significant (p>0.05) difference between control group and treatment group (Figure 2).

## Discussion

*Achillea millefolium* known as yarrow is used as an appetizer and treatment of dyspepsia symptoms in traditional medicine of various countries(Saeidnia et al., 2011 ▶). In the present study, it was found that orexigenic effect of yarrow extract was dose-dependent. The extract at the concentrations of 50 and 100mg/kg could increase the amount of food intake by rats during 2 hr after gavage, while at the concentration of 150mg/kg, the extract showed no effect on appetite during the first 6 hr after gavage and reduced the amount of food intake after that. The reason for decrease in appetite after administration of 150mg/kg of extract is not clear. It may be due to some side effects of *A. millefolium* at high doses.

Few studies on the orexigenic effects of yarrow have shown that it improves food intake by broiler chickens as a measure of their growth (Rhiannon 2005 ▶). Also, Cross and colleagues added 10g/kg powdered yarrow to the daily diet of broiler chicks and measured their weekly food consumption. As a result, their food consumption and performance was increased (Cross et al., 2007 ▶).

Matsumura and his colleagues conducted a study on herbal mixture of Rikkunshito which is known as an appetizer and reduces dyspepsia symptoms in Japanese Traditional Medicine (Matsumura et al., 2010 ▶). Rikkunshito powder was added to rat’s drinking water at different concentrations. Following this intervention, the expression of ghrelin mRNA was increased in stomach. One of the main components of *A. millefolium *is flavonoids (Saeidnia et al., 2011 ▶) . Takeda and his colleagues suggested the role of flavonoids in appetizing effect of Rikkunshito (Takeda et al., 2008 ▶). These flavonoids blocked serotonin receptors *in vitro*which caused an increase in plasma ghrelin levels (Fujitsuka et al., 2009 ▶). So far, there is no *in vivo* study investigating the flavonoids effect on plasma ghrelin levels.

It was shown that after administration of ghrelin to rats, their appetite was improved(Asakawa et al., 2001 ▶). Thereafter, ghrelin was known as an appetizing peptide. In addition, there is a correlation between plasma ghrelin levels and reduction of symptoms in patients with dyspepsia. Therefore, ghrelin and its analogues could be useful in these patients (Akamizu et al., 2010a ▶).

In the second part of our study, the hydro-alcoholic extract of yarrow reduced plasma ghrelin levels significantly in the first hour but later, there was no significant difference in plasma ghrelin levels compared with control group. In other words, yarrow could not increase plasma ghrelin levels and therefore it did not increase food intake at the dose of 100mg/kg.

According to a study performed by Cummings et al. on 10 healthy subjects, plasma ghrelin level was reduced one hour after food intake(Cummings et al., 2001 ▶). In another study on the subjects who fasted over the night, plasma ghrelin level was significantly reduced 1-2 hours after food intake (Tschop et al., 2001 ▶). Therefore, it can be concluded that other mechanisms may have roles in orexigenic effect of yarrow. As an additional mechanism, secretion of hormones and gastrointestinal peptides can be mentioned. Stimulation of gastric enzyme secretions by sesquiterpenes of yarrow was a mechanism for increased food intake (Rhiannon 2005 ▶). According to Konturek and his colleagues, a fall in plasma ghrelin level after food intake can increase the pulsatile exocrine secretions of pancreas (Konturek et al., 2003 ▶). Even though, yarrow could not increase the plasma ghrelin level, the reduction observed in the first one hour after extract administration could denote the stimulation of secretion of pancreatic enzymes.

As mentioned previously, plasma ghrelin level is higher in fasting state compared to non-fasting state, but this is not successive. Pursuant to a study by Natalucci and his colleagues, 24-hr ghrelin profile in the body has a dual rhythm i.e. high levels in the morning and low levels at night. Moreover, it follows a pulsatile pattern. In healthy subjects who received the usual meals, 8 ghrelin concentration peaks were seen in 24 hr. So, ghrelin had circadian and pulsatile profile (Natalucci et al., 2005 ▶). In our study, there was an increase in plasma ghrelin level within 2 hr after sampling, but after a peak concentration in the second hour, the plasma ghrelin level reduced.

According to this study and those previously mentioned,* A. millefolium* administration improved the daily energy intake. Also, yarrow is suggested for the treatment of dyspepsia in traditional medicine. Hence, in the present study along with investigating the effect of yarrow on appetite, we evaluated the changes in plasma ghrelin levels after administration of orexigenic doses of hydro-alcoholic extract of plant, which is regarded as a probable mechanism.

In this study, it was observed that hydro-alcoholic extract of *A. millefolium* at doses of 50 and 100 mg/kg could increase the amount of energy intake by rats in 24 hr, but at the dose of 150mg/kg, it had an inverse effect on the appetite of rats and consequently their food intake. As a conclusion, *A. millefolium* can increase food intake even though further investigations are required. Returning to the question posed at the beginning of this study, it is now possible to state that orexigenic mechanism of *A. millefolium* is not mediated by increase in ghrelin level. 
